# Beat Perception and Sociability: Evidence from Williams Syndrome

**DOI:** 10.3389/fpsyg.2016.00886

**Published:** 2016-06-20

**Authors:** Miriam D. Lense, Elisabeth M. Dykens

**Affiliations:** ^1^Marcus Autism Center, Children’s Healthcare of Atlanta, Emory University, AtlantaGA, USA; ^2^Vanderbilt Kennedy Center, Vanderbilt University Medical Center, NashvilleTN, USA; ^3^Program for Music, Mind and Society, Department of Otolaryngology, Vanderbilt University Medical Center, NashvilleTN, USA

**Keywords:** Williams syndrome, beat, rhythm, social, communication, meter

## Abstract

Beat perception in music has been proposed to be a human universal that may have its origins in adaptive processes involving temporal entrainment such as social communication and interaction. We examined beat perception skills in individuals with Williams syndrome (WS), a genetic, neurodevelopmental disorder. Musical interest and hypersociability are two prominent aspects of the WS phenotype although actual musical and social skills are variable. On a group level, beat and meter perception skills were poorer in WS than in age-matched peers though there was significant individual variability. Cognitive ability, sound processing style, and musical training predicted beat and meter perception performance in WS. Moreover, we found significant relationships between beat and meter perception and adaptive communication and socialization skills in WS. Results have implications for understanding the role of predictive timing in both music and social interactions in the general population, and suggest music as a promising avenue for addressing social communication difficulties in WS.

“When you play the guitar, do you use a metronome or do you use your heart to keep the beat?”

-question posed to a songwriter by an individual with Williams syndrome attending a music camp

## Introduction

Williams syndrome (WS) is a genetic, neurodevelopmental disorder caused by the deletion of ∼28 genes on chromosome 7 (Ewart et al., 1993). WS is associated with a unique cognitive behavioral profile including mild to moderate cognitive impairment, greater verbal than spatial abilities, anxiety, and hypersociability (see Martens et al., 2008 for a review). Additionally, people with WS have pronounced auditory sensitivities and increased emotional responsiveness to music, even as they vary greatly in their musical skills (Lense et al., 2013). A better understanding of the musical profile in WS may help determine how their musical interests and abilities fit with other aspects of the WS phenotype, as well as lead to insights into gene-brain-behavior relationships involved in musical engagement. One area of particular interest is skills related to rhythm and timing, which are crucial in both music and social communication and interaction (e.g., Patel, 2008).

Many aspects of timing are incorporated into music including tempo, beat, rhythmic patterns, meter, and temporal variability or expressive timing (Honing, 2013). Rhythm refers to the pattern of durations of the musical notes. Rhythm is often perceived within the framework of a musical beat, i.e., a regular pulse marking equally spaced time intervals (Large and Palmer, 2002). The structure provided by a regular, predictable beat enhances rhythm discrimination and rhythm production abilities (e.g., Essens, 1986; Patel et al., 2005; Grahn and Brett, 2009). Additionally, the beat can be organized into different hierarchical levels of strong and weak beats, which make up the musical meter (for example the strong-weak beat pattern of a march versus the strong-weak-weak beat pattern of a waltz) and further enhance beat and rhythm perception and production (e.g., Grahn and Brett, 2007; Grube and Griffiths, 2009). Tempo refers to the pace or rate of the music, i.e., the speed of the musical beats. Temporal variability refers to the timing differences expressed in a musical performance, which might be perceived as a more mechanical versus expressive production of the music.

Most studies of musical timing in WS have focused on perception of rhythmic patterns, with variability evident both within and across studies. For example, several studies have used the Gordon Primary Measures of Music Audiation (PMMA; Gordon, 1986) to examine rhythmic abilities. In this task, participants make same/difference judgments regarding the rhythm of two consecutive musical phrases (i.e., rhythm discrimination judgments). One study reported that adolescents with WS performed worse than chronological age-matched typically developing (TD) peers on this task (Hopyan et al., 2001); indeed, the performance of the WS group did not differ from chance. Using the same rhythm task, Don et al. (1999) reported that children and adolescents with WS performed at a level generally consistent with their receptive vocabulary skills (used to estimate verbal mental age), suggesting that musical and simple language skills may both be areas of relative strength in WS. At the same time, individuals with WS demonstrated worse rhythm perception skills than TD individuals of a similar verbal mental age (who had fewer years of musical exposure). In contrast, Levitin (2005) reported that their participants with WS performed equivalently to highly trained TD music students on these rhythm discrimination items, though formal analyses were not presented. Finally, Martens et al. (2010) also reported poorer performance on a similar rhythm discrimination task in WS compared with same-aged TD peers. Variable findings across studies may relate to differences in age of participants (children versus adults), type of control group (mental age versus chronological age matched, who also have different years of musical exposure and training), and recruitment site (music camp versus clinic). Even so, this work suggests that rhythm discrimination skills are not necessarily preserved in WS though may be commensurate with language skills.

However, as noted by Levitin et al. (2004), results may also have been affected by task order: participants in Don et al. (1999) and Hopyan et al. (2001) completed the rhythm task after a melody task and attention difficulties in WS may have led to decreased performance as the assessments progressed. Levitin et al. (2004) also suggest that small manufacturing defects in the PMMA test stimuli might have disproportionately affected the participants with WS given their auditory hypersensitivities. Finally, these same/difference tasks require holding the original musical phrase in memory to compare with the second phrase. None of the studies controlled for auditory working memory, despite findings that individuals with WS may show poorer working memory than expected based on receptive language skills (Don et al., 1999; Rhodes et al., 2011).

There is also limited data on musical beat and rhythm production skills in WS. In a sample of 25 children and adults with WS, Martens et al. (2010) reported that participants performed as well as chronological age-matched TD controls on a task that required them to clap in time to the beat of musical passages, but performed worse than TD controls at reproducing rhythmic patterns by clapping or singing them. Levitin and Bellugi (1998) found that eight individuals with WS were able to clap back rhythmic patterns based on the PMMA as well as younger TD children (who were not strictly matched on mental age or musical training). Additionally, the errors made by the WS group tended to be musically compatible (i.e., fit within the overarching beat and metrical structure of the phrase). Thus, there is some evidence that individuals with WS show strengths in beat-based production tasks, while findings for specific rhythmic pattern reproduction are unclear. Differences in task designs and task order, perceived musical nature of the stimuli, and testing set-up may also contribute to these differences. For example, in the Levitin and Bellugi (1998) study, participants with WS clapped rhythms in response to those clapped by another person while the stimuli in Martens et al. (2010) were prerecorded. Given the hypersociability in WS, a social context could lead to differential performance results. Indeed, Levitin et al. (2004) reported improved performance in individuals with WS on a modified PMMA task when stimuli were presented in person rather than a recording. Assessing rhythm in a more naturalistic musical context may also impact findings as it may be more engaging and better maintain participants’ attention and interest.

Overall, the results of previous studies examining aspects of rhythm and timing in WS present a somewhat mixed set of results. Moreover, these studies have been limited by small sample sizes (8–20 individuals with WS) and task demands (for example, working memory load, attention requirements, potential stimuli defects in the PMMA). Studies have also primarily focused on the rhythmic aspect of music with limited attention given to other crucial aspects of musical timing – beat and meter – that provide a framework for structuring musical rhythms. The clapping tasks used by Levitin and Bellugi (1998) and Martens et al. (2010) suggest that individuals with WS are able to execute motoric responses in line with the musical beat. Therefore, studies that explicitly examine meter and beat perception in WS are needed to better understand the musical profile in WS. Additionally, previous studies have not fully examined individual differences in these abilities despite the variability both within and across studies.

Beat and meter perception are particularly relevant for WS when considering the WS social profile. A hallmark of WS is hypersociability, including increased empathy and motivation to interact with others (Jarvinen and Bellugi, 2013; Thurman and Fisher, 2015). Intriguingly, beat perception in music creates a salient signal for perceptual and motor entrainment (e.g., Large and Jones, 1999; Patel et al., 2005; Fujioka et al., 2012). These types of synchronized behaviors are linked with social bonding and prosocial behaviors from infancy through adulthood in TD populations. For example, adults who engage in synchronized musical activities show increased cooperation even at their own expense (Anshel and Kippler, 1988; Wiltermuth and Heath, 2009), and children are more helpful to each other following musical versus non-musical games (Kirschner and Tomasello, 2010). Infants are more helpful to an experimenter who has synchronously bounced with them to the beat of music versus an experimenter who bounced asynchronously (Cirelli et al., 2014). The ability of music to engender synchronous activities and increase social cohesion has been proposed as an adaptive value of music and may explain its ubiquity in social situations such as maternal-infant interaction, religious ceremonies, sports events, and military activities (Trevarthen et al., 1999–2000; Cross, 2003; Dissanayake, 2008; Wiltermuth and Heath, 2009). Additionally, numerous studies have documented behavioral and neural overlap in beat and rhythm processing in music and language in typical populations (e.g., Tierney and Kraus, 2013; Magne et al., 2016) and atypical populations with reading and/or language impairments (e.g., Corriveau and Goswami, 2009; Cumming et al., 2015). Indeed, beyond the potential social adaptive values of perceiving a beat in music, Patel (Patel, 2006; Patel et al., 2009) has hypothesized that human’s abilities to perceive and synchronize to a beat may be due in part to our status as vocal learners, perhaps in combination with factors such as being able to engage in non-vocal movement imitation and living in complex social groups.

Though, WS is characterized by increased social motivation and relatively stronger simple verbal skills, recent research implicates an uneven linguistic and social profile in WS (Mervis and Becerra, 2007). Of particular relevance for beat and rhythm processing, onset of language is delayed in WS, which appears to be related to motor delays (Masataka, 2001). Additionally, similar to those with language impairments, individuals with WS show difficulties using stress-patterns for word meaning (Plesa Skwerer et al., 2007). Moreover, difficulties in social communication and interpersonal interactions are common in WS including impairments in initiating and maintaining appropriate conversations (Laws and Bishop, 2004; Stojanovik, 2006). These difficulties lead to difficulties forming and maintaining relationships with peers (see Thurman and Fisher, 2015 for a review).

Thus, the vast individual variability in musical and social behaviors in WS provides an opportunity to examine relationships between musical and social communication behaviors. This may provide a novel window into understanding the role of timing in both music (e.g., beat, meter processing) and social communication (e.g., stress prosody; back-and-forth rhythm of a conversation; anticipating social cues). Previous research has indicated increased links between musical and social emotions in WS compared to TD populations (Ng et al., 2013; Lense et al., 2014b; Pridmore et al., 2014) and musical engagement is sometimes used as a vehicle for social engagement in WS (for example, as described in Levitin and Bellugi, 1998; Levitin et al., 2004). However, direct links between music and social behaviors within the realm of beat perception are unexplored.

In this two-part study, we first examined musical beat and meter perception in a large sample of individuals with WS, and identified how these perceptions related to cognitive abilities, musical training, and musical exposure. We also examined how individual differences in auditory processing style (i.e., reliance on the fundamental frequency versus harmonic overtones) predicted beat and rhythm skills. We specifically chose well-validated tasks that did not have a working memory requirement (i.e., same/difference judgments) and examined beat and meter perception in the context of actual musical examples rather than isolated rhythmic excerpts. Additionally, we examined how differences in the stimuli (for example, tempo, genre, beat variability) predicted individuals’ performance with the stimuli. Thus, we were able to examine both how characteristics of participants and characteristics of stimuli impacted the individual differences in beat perception in WS. We hypothesized that individuals with WS versus TD controls would show poorer beat perception abilities but significant individual variability, and would also benefit more from music that had a consistent beat and that was of a familiar genre.

In the second study (which included a subset of individuals from Study 1, as well as new participants), we conducted exploratory analyses on the relationship between musical beat, meter perception and adaptive social communication skills in WS. Adaptive skills are behaviors that people routinely perform to meet the personal and social demands of daily life. We hypothesized that greater beat and meter perception skills would be associated with improved adaptive communication and social skills, consistent with beat perception scaffolding entrainment of social engagement and interaction.

## Study 1: Individual Differences in Beat and Meter Perception

### Methods

#### Participants

Participants included 74 children and adults (mean age: 26.4 ± 9.6, 50.0% male) with genetically confirmed diagnoses of WS who were recruited from a residential summer camp or national convention. Both children and adults were recruited as we aimed to identify if age accounted for individual differences in beat perception. Due to exclusion of invalid data or changes in study protocol over the multi-year study period, some participants completed both meter and beat perception tests (*n* = 57) while others completed only one of the tests (Beat = 59; Meter = 72).

The beat and meter tests were also administered to a comparison group of 53 TD participants (mean age: 24.3 ± 9.4, 48.1% male); 35 of them completed both the beat and meter tests while an additional 18 completed only the meter test. As shown in **Table 1**, WS and TD participants were well-matched on age, sex, types of musical training, cumulative years of individual lessons, percent with percussion/piano training, and time spent currently listening to music. On average, however, the WS group spent more time currently playing music. As expected, the TD versus WS participants had significantly higher IQ scores.

**Table 1:** Demographic information for Study 1.

	WS (*n* = 74)	TD (*n* = 52)	*p*-value
Age (years)	26.4 ± 9.6	24.3 ± 9.4	0.234
Sex (% male)	50	48.1	0.832
IQ	70.0 ± 14.4	106.9 ± 11.9	<0.001
Types of musical training	2.79 ± 1.9	2.2 ± 1.7	0.074
Cumulative years of individual extra-curricular music lessons	5.8 ± 8.6	4.04 ± 6.8	0.229
Piano/drum individual training (%)	44.6	44.2	0.964
Time currently play music (hrs)	1.2 ± 1.9	0.5 ± 1.0	0.011
Time currently listen to music (hrs)	3.1 ± 2.5	3.2 ± 3.3	0.896


The University Institutional Review Board approved the study, and written, informed consent was obtained from TD adult participants, and from the parents/guardians of participants with WS and TD minor participants. Participants with WS and TD minors provided verbal and written assent.

#### Measures and Procedures

##### Musical Background

Typically developing participants and parents of participants with WS completed a Musical Background Questionnaire (Lense and Dykens, 2012; Lense et al., 2013). Consistent with previous research (Lense et al., 2013, 2014a), musical training was quantified both as the number of types of formal music lessons received (including individual and group lessons both within school or as an extra-curricular activity, as well as ensemble participation) and as the cumulative duration of individual extra-curricular music lessons. The former appears to better reflect musical training experiences in WS while the latter is a standard metric used in TD studies.

#### Behavioral Assessments

##### Cognitive Assessment

Participants were individually administered the Kaufman Brief Intelligence Test, 2nd edition (KBIT-2; Kaufman and Kaufman, 2004), which provides verbal, non-verbal, and full-scale IQ scores. The full-scale IQ was used as an index of cognitive abilities.

##### Beat Alignment Test (BAT)

A subset of 16-items from the Beat Alignment Test, version 2 (BAT: Iversen and Patel, 2008) was used to assess beat perception. Participants listened to music from different genres (rock, jazz, or orchestral pops) with a superimposed track of beeps that either aligned or misaligned with the musical beat. On misaligned tracks, beeps were phase shifted by 30% ahead of or behind the beat. Participants were given demonstration items with feedback prior to the test, and then responded if the beeps matched the beat of the music. Although standard administration asks that participants stay still while completing the BAT, many individuals with WS were unable to inhibit their motoric response (e.g., rocking, head shaking, or tapping hand or foot). BAT scores were converted to d’ to control for response biases (i.e., a tendency to answer that beeps matched versus did not match the beat). A metric used in signal detection theory (Green and Swets, 1966), d’ is computed as the z-standardized hit rate (correctly responding that beeps matched the beat) minus the z-standardized false alarm rate (incorrectly responding that beeps matched the beat when they did not match). In this way, d’ provides a measure of perceptual sensitivity that considers both accuracy and response bias. The BAT stimuli were presented at ∼68 dB from either two speakers approximately 40 cm in front of the participant or from one speaker ∼60 cm above their head. A subset of 14 participants with WS completed the BAT a second time over a 1–3 years period with a test–retest reliability ICC = 0.71.

##### Montreal Battery of Evaluation of Amusia Meter subtest (MBEA-m)

The meter subtest is one of six subtests of the Montreal Battery of Evaluation of Amusia (MBEA; Ayotte et al., 2002; Peretz et al., 2003), a widely used measure for assessing musical perception skills. Participants were presented with 30 two-phrase harmonic melodies in a piano timbre, with chords emphasizing either duple or triple meter. Following each melody, participants determined if the melody was “in 2” (march) or “in 3” (waltz). Participants were given practice examples with feedback prior to the test items and shown visuals of march/in 2 and waltz/in 3. In contrast to the BAT, participants were encouraged to clap, tap, sing, or otherwise move to feel the beat. (During example items, the experimenter demonstrated moving to a march versus a waltz to encourage the use of different movements in determining the meter.) Participants’ total scores (out of 30) were used as a measure of meter perception. MBEA-m stimuli were presented at 68 dB from two speakers approximately 40 cm in front of the participant. A subsample of 21 individuals with WS completed the MBEA a second time following a 1–3-years delay. Test–retest reliability was excellent (ICC = 0.820) based on Fleiss (1986) guidelines.

##### Sound Perception

To assess the sound processing style of participants with WS, the 12-items version of the spectral-fundamental processing test (SFP; Schneider et al., 2005; Wengenroth et al., 2010) was administered. The SFP characterizes an individual’s dominant auditory processing style along a continuum from primary spectral processing (perceive sound by decomposing it into its harmonics) to primary fundamental processing (perceive sound based on the fundamental frequency). On the SFP, participants heard pairs of 500-ms tones repeated twice. The tones varied in number, height and averaged frequency of their harmonics (see Wengenroth et al., 2010 for a full description). Participants reported if the second tone in the pair was higher or lower than the first. For each tone pair, the perceived direction of pitch change reflected either spectral or fundamental processing. An SFP index was computed [(number of spectrally perceived items – number of fundamentally perceived items)/total number of items] where scores vary from -1 to + 1. Higher scores reflect greater use of spectral processing and lower scores reflect greater use of fundamental processing. Scores around 0 reflect no consistent preference.

#### Analyses

Consistent with prior research, the MBEA-m scores were significantly negatively skewed while the BAT scores were more normally distributed in both the TD and WS groups. Therefore, we used non-parametric statistics to compare performances between the WS and TD groups, and to examine correlations between MBEA-m and BAT performance with age, IQ, musical training (number of types of lessons and cumulative duration of individual lessons), time spent playing and listening to music, and sound processing style in the WS participants (sound processing was not collected in TD participants). Variables that were significantly related to MBEA-m and BAT scores were then entered into linear regression analyses to assess their unique contributions to variance in beat and meter perception skills in WS. Parametric linear regressions were appropriate given the normally distributed residuals.

Preliminary analyses of the BAT data suggested that for both WS and TD participants, some items were easier than others. As the BAT stimuli reflect real musical excerpts, we examined how certain stimuli-specific factors might have influenced performance in the WS and TD groups. We used multilevel logistic models that allowed us to cluster items within individual participants, thus controlling for the individual participant variability in BAT performance. We examined musical genre (rock versus jazz versus orchestral pops, with rock music used as the reference); tempo (based on the inter-onset-intervals of the beat (in ms), grand-centered); and beat variability (based on the coefficient of variability (CV; Lovie, 2005) of the inter-onset-intervals, grand-centered) as predictors of item-level performance in WS and TD groups.

### Results

#### Beat Alignment Test

Beat Alignment Test performance was highly variable in the WS and TD groups. Overall, TD participants demonstrated significantly greater performance on the BAT than WS participant (d’ = 2.17 ± 0.75 versus 1.37 ± 0.98, Mann–Whitney *U* = 540.5, *p* < 0.001). In WS, BAT performance was associated with IQ (ρ = 0.445, *p* < 0.001), types of musical training (ρ = 0.320, *p* = 0.016), cumulative years of individual music lessons (ρ = 0.357, *p* = 0.007), and sound processing style (ρ = -0.401, *p* = 0.002) but not with age or time spent playing or listening to music. The negative correlation between BAT and sound processing style suggests that greater BAT performance is associated with greater use of a fundamental processing style. In the TD group, BAT performance was similarly associated with types of musical training (ρ = 0.372, *p* = 0.03) and cumulative years of individual music lessons (ρ = 0.360, *p* = 0.036) but not with IQ, age, or time spent playing or listening to music.

The regression analysis including the four predictor variables (IQ, sound processing, and the two musical training variables) explained 28.7% (adjusted *R*^2^) of the variance in BAT score [*F*(4,51) = 6.523, *p* < 0.001] in the WS group (**Table 2**). IQ and sound processing were the strongest predictors of BAT score, explaining 7.6 and 11.0% of the variance in BAT scores, respectively. Cumulative duration of independent musical training predicted an additional 3.8% of the variance.

**Table 2:** Regression model predicting BAT performance in WS.

Predictor	β	*t*	*p*	Semipartial *r*
(Constant)		-0.7	0.487	
*IQ*	*0.285*	*2.417*	*0.019*	*0.275*
*Sound processing style*	-*0.349*	-*2.909*	*0.005*	-*0.331*
Types of musical training	0.026	0.163	0.871	0.019
Duration of individual music training	0.261	1.712	0.093	0.195


*Significant predictors are italicized.*

The multilevel binomial models confirmed the significant individual variability in BAT performance in both the WS [χ^2^(58) = 141.31, *p* < 0.001] and TD groups [χ^2^(34) = 66.69, *p* < 0.001]. Results of the models can be found in **Table 3** (WS) and **Table 4** (TD). When examining effects of genre, tempo, and beat variability, only beat variability predicted item level performance in the WS group. Each percent increase in beat variability (compared with average beat variability across stimuli) was associated with a 70.4% chance of correctly answering an item on the BAT [*t*(881) = -4.599, *p* < 0.001; Odds Ratio = 0.78]. However, further analysis found that this was due to one particular excerpt, an orchestral pops version of the Superman theme. The beat of this stimulus was more than twice as variable (CV = 7.8%) as the next most variable stimulus (an orchestral pops rendition of Richard Rogers waltzes CV = 3.7%) or the average of all other stimuli included in our test battery (CV = 2.7%). When this item was excluded, beat variability was no longer a significant predictor of item accuracy. Instead, orchestral pops stimuli (versus rock stimuli) were associated with only a 71.3% chance of a correct answer [*t*(761) = -2.049, *p* = 0.041, OR = 0.62] when controlling for tempo and beat variability.

**Table 3:** Multilevel model predicting item accuracy on the BAT in WS (*n* = 59).

Predictor	β	*t*	*p*	Semipartial *r*
(Constant)		5.595	<0.001	
*IQ*	*0.247*	*2.287*	*0.026*	*0.240*
*Sound processing style*	-*0.224*	-*2.052*	*0.044*	-*0.215*
Types of musical training	0.283	1.853	0.069	0.194
Duration of individual music training	0.069	0.469	0.641	0.049


*Significant predictors are italicized.*

**Table 4:** Multilevel model predicting item accuracy on the BAT in TD (*n* = 35).

Fixed effect	Coefficient	*SE*	*t*-ratio	df	*p*	Odds ratio	Confidence interval
Intercept	1.11	0.16	6.827	58	<0.001	3.03	(2.192, 4.203)
Tempo (ms)	-0.000056	0.000612	-0.092	881	0.926	0.99	(0.999, 1.001)
*Beat variability (CV)*	-0*.24*	*0.05*	-*4.599*	*881*	*<0.001*	*0.79*	*(0.708, 0.871)*
Jazz genre	0.037	0.17	0.217	881	0.828	1.04	(0.542, 1.261)
Pops genre	-0.19	0.22	-0.887	881	0.375	0.83	(0.542, 1.261)


*Tempo and beat variability are grand-centered around the mean. The reference genre is rock music.*

In contrast, in TD participants, tempo and jazz genres were significant predictors of accuracy. A 1 ms increase in inter-onset-interval of the beat (i.e., slower tempo) was associated with a 90.8% chance of correctly answering a BAT item while jazz stimuli (versus rock stimuli) were associated with an 83% chance of correctly answering a BAT item.

#### Montreal Battery of Evaluation of Amusia Meter Subtest

**FIGURE 1:**
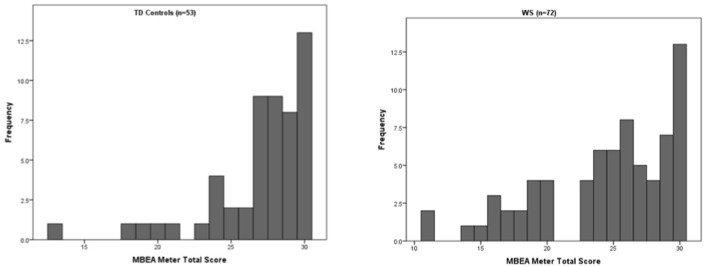
**Distribution of MBEA-m scores in TD (left) and WS (right)**.

As depicted in **Figure 1**, there were vast individual differences in meter perception in both the WS and TD groups though overall performance was significantly greater in the TD (mean = 26.92 ± 3.5, median = 28) than WS (mean = 24.43 ± 5.1, median = 26) group (Mann–Whitney *U* = 1333, *p* = 0.004). Among WS participants, greater MBEA-m performance was associated with higher IQ (ρ = 0.342, *p* = 0.003), number of lesson types (ρ = 0.413, *p* < 0.001), and cumulative years of musical training (ρ = 0.424, *p* < 0.001). MBEA-m performance was also associated with sound processing style (ρ =- 0.287, *p* = 0.014), indicating that individuals with a greater fundamental processing style had better meter perception skills on the MBEA-m. Among TD participants, greater MBEA-m performance was associated with number of lesson types (ρ = 0.388, *p* = 0.004), cumulative years of musical training (ρ = 0.335, *p* = 0.004), and time spent currently playing music (ρ = 0.284, *p* = 0.041). No other significant relationships were found.

The regression analysis including IQ, sound processing, and the two musical training variables predicted 25.3% (adjusted *R*^2^) of the variance in MBEA-m performance in WS [**Table 5**; *F*(4,64) = 6.757, *p* < 0.001]. IQ and sound processing had the greatest predictive value on MBEA-m performance, accounting for approximately 5.8 and 4.6% of the variability in MBEA-m performance when controlling for the other factors, with types of musical training accounting for an additional 3.8% of variance.

**Table 5:** Regression model predicting MBEA-m performance in WS.

Fixed effect	Coefficient	*SE*	*t*-ratio	df	*p*	Odds ratio	Confidence interval
Intercept	2.29	0.33	6.931	34	<0.001	9.91	(5.058, 19.424)
*Tempo (ms)*	-0*.002449*	*0.00086*	-*2.864*	*521*	*0.004*	*0.99*	*(0.996, 0.999)*
Beat variability (CV)	-0.12	0.078	-1.495	521	0.136	0.89	(0.763, 1.038)
*Jazz genre*	-*0.71*	*0.29*	-*2.406*	*521*	*0.016*	*0.49*	*(0.275, 0.878)*
Pops genre	-0.29	0.45	-0.651	521	0.515	0.74	(0.305, 1.817)


*Tempo and beat variability are grand-centered around the mean. The reference genre is rock music.*

#### Beat Alignment Test and Montreal Battery of Evaluation of Amusia Meter

Among the 57 participants with WS with both BAT and MBEA-m scores, performance on the two measures was highly correlated (ρ = 0.610, *p* < 0.001). This is an expected finding, as meter perception emerges from the hierarchical organization of beats. Therefore, we conducted a step-wise regression examining MBEA-m performance with BAT performance. At Step 1, we entered the significant predictors from the original analysis to confirm similar results on MBEA-m performance in this subset of participants (IQ, sound processing, and the two musical training variables). Results were similar to findings with the full set of participants with MBEA-m data, explaining 27.9% of the variance in MBEA-m performance in this smaller sample. As before, IQ (β = 0.263, *t* = 2.207, *p* = 0.032, s*r*^2^ = 6.5%) and sound processing style (β = -0.277, *t* = -2.273, *p* = 0.027, s*r*^2^ = 6.9%) were the greatest predictors of MBEA-m performance. However, the addition of BAT d’ score at Step 2 explained an additional 8.8% (adjusted *R*^2^ = 36.7%) of the overall MBEA-m variance and BAT d’ score was the only significant predictor (β = 0.378, *t* = 2.825, *p* = 0.007, s*r*^2^ = 9.4%). IQ and sound processing style (which had predicted BAT performance) were no longer unique predictors of MBEA-m once beat perception abilities were taken into account. (Results were similar when the Superman item was excluded when calculating BAT d’ scores.)

## Study 2: Beat and Meter Perception and Social Communication Skills

### Methods

#### Participants

Data for Study 2 were collected from 50 adults with WS who attended a 1-week residential summer camp over a 4-years period. The camp focused on musical activities such as songwriting and performances, as well as developing social and daily living skills. Musical talent was not required to attend this program and attendees varied widely in their music abilities, as reflected in Study 1 and previous work (e.g., Lense and Dykens, 2012; Lense et al., 2013). Of the 50 participants in Study 2, 31 also participated in Study 1 and 19 were new participants.

Due to changes in study protocol across years, 37 (mean age: 26.2 ± 8.4 years, 56.8% male) of the 50 participants completed the beat test (BAT) and 40 (mean age: 26.8 ± 8.3 years, 64.0% male) completed the meter (MBEA-m) test, with 28 of these participants completing both tests. One individual who completed the MBEA-m was excluded from the BAT because they did not follow task directions. An additional participant was excluded from both BAT and MBEA testing because they did not understand directions. Results were very similar when analyses only included participants with both tests. Demographic information for these groups of participants is provided in **Table 6.**

**Table 6:** Demographic information, Vineland-II, and BAT/MBEA-m scores for Study 2.

	Participants with BAT (*n* = 37)	Participants with MBEA-m (*n* = 40)
Age (years)	26.2 ± 8.4	26.8 ± 8.3
Sex (% male)	56.8	65.0
IQ	70.4 ± 13.5	71.3 ± 15.2
Types of musical training	2.9 ± 1.8	3.3 ± 1.8
Cumulative years of individual extra-curricular music lessons	6.6 ± 9.3	7.6 ± 9.7
Vineland-II communication	60.9 ± 15.9	65.2 ± 17.2
Vineland-II daily living skills	61.5 ± 8.3	63.6 ± 9.6
Vineland-II socialization	72.4 ± 7.1	72.8 ± 8.2
BAT d’	1.5 ± 1.2	–
MBEA meter	–	24 ± 5.6


#### Measures

Participants completed the KBIT-2, BAT, and MBEA-m, described in Study 1. Adaptive functioning was assessed with the Vineland Adaptive Behavior Scales, 2nd edition (Vineland-II; Sparrow et al., 2005), which identifies adaptive functioning in three domains: Communication, Daily Living Skills, and Socialization. This semi-structured, standardized interview was conducted over the phone with participants’ primary caregiver. The three domains yield standard scores (*M* = 100, *SD* = 15), which were used in analyses.

#### Analyses

We conducted zero-order and partial correlations controlling for IQ between the BAT, MBEA-m, and Vineland-II domain scores.

### Results

#### Beat Alignment Test

**FIGURE 2:**
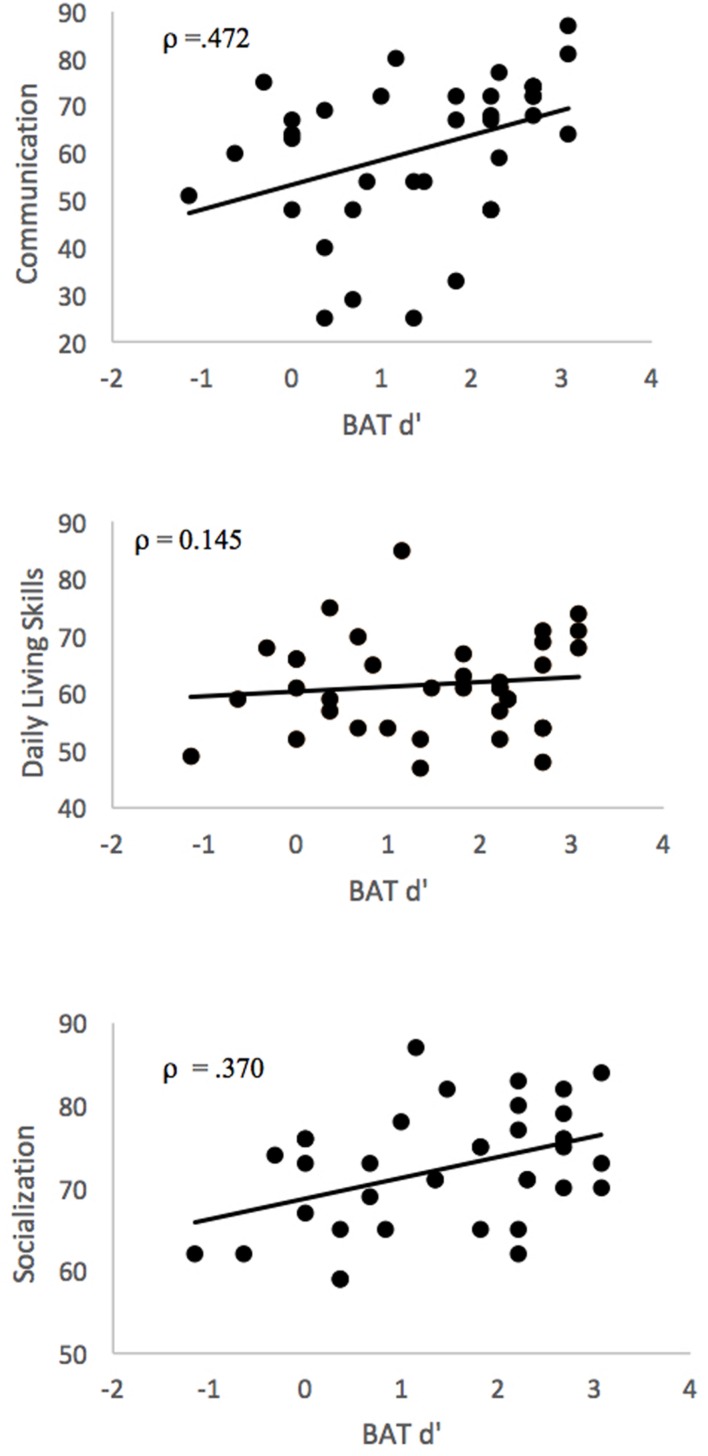
**Correlations between BAT d’ and Vineland-II scores**.

**Figure 2** depicts the relationship between BAT scores and Vineland-II scores. BAT performance (d’) was significantly associated with Vineland-II Communication (ρ = 0.472, *p* = 0.003) and Socialization (ρ = 0.370, *p* = 0.024) but not Daily Living Skills (ρ = 0.145, *p* = 0.391). This pattern of findings remained when controlling for IQ (Communication: ρ = 0.436, *p* = 0.008, Socialization: ρ = 0.326, *p* = 0.052, Daily Living Skills: ρ = 0.141, *p* = 0.411).

#### Montreal Battery of Evaluation of Amusia Meter Subtest

**FIGURE 3:**
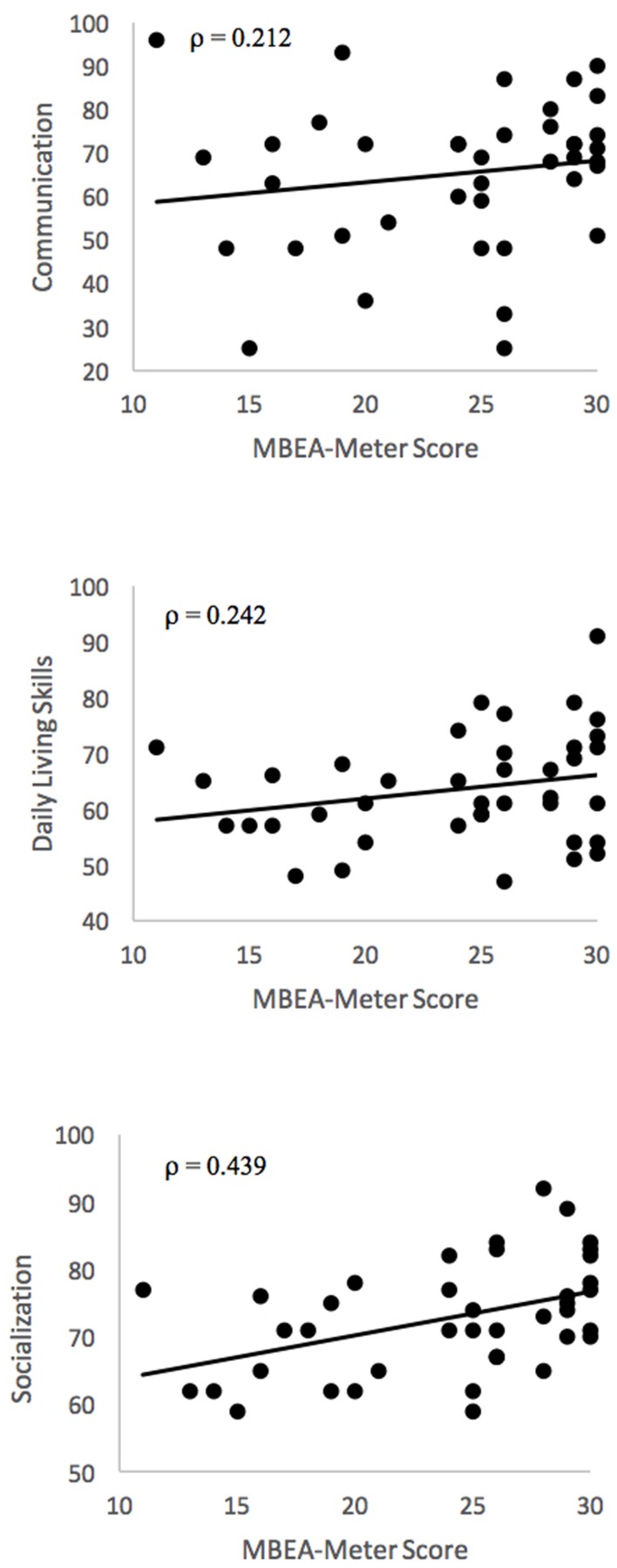
**Correlations between MBEA-m and Vineland-II scores**.

As depicted in **Figure 3**, MBEA-m scores were associated with the Vineland-II Socialization domain (ρ = 0.439, *p* = 0.005), even when controlling for IQ (ρ = 0.307, *p* = 0.057), but MBEA-m performance was not associated with Communication or Daily Living Skills (ρ’s = 0.21 and 0.24, respectively).

## Discussion

In contrast to literature portraying preserved musical skills in WS, this study instead highlights considerable variability in beat and meter perception skills in people with this syndrome. On average, individuals with WS have poorer beat and meter perception skills than age-matched typically developing individuals with comparable musical training. Beat and meter perception was influenced by individual-level characteristics (e.g., IQ, sound processing style, musical training), as well as stimuli-level characteristics (e.g., musical genre and beat variability).

While our findings may seem inconsistent with two previous reports suggesting age-appropriate beat abilities in WS to clap to/in response to music, there are several possibilities for these differences in findings including task demands, constructs, and sample characteristics. For example, the current study examined perceptual abilities while the previous studies documented production skills. Additionally, the perception tasks in the current study required explicit answers while the clapping tasks in the production studies may have been more implicit. Indeed, Levitin and Bellugi (1998) noted that in their clapping tasks, both WS and TD participants naturally responded to the experimenter’s clapping by clapping back in time themselves (i.e., implicitly preserving the overall meter). Thus, it is possible participants with WS do better on tasks that do not require explicit responses. This pattern of greater impairment in explicit versus implicit musical tasks has previously been seen in cases of otherwise TD individuals with extremely poor pitch perception abilities (Loui et al., 2008). In contrast, studies of beat perception in TD groups have documented cases where a given individual’s perceptual abilities are stronger than their ability to tap to the beat (Iversen and Patel, 2008). Future studies will need to directly assess beat perception and production skills in the same individuals with WS to examine relationships between these abilities and to determine if some individuals have significant difficulties specifically on explicit perception tasks. Martens et al. (2010) tested participants with WS on a variety of melodic and rhythmic perception and production tasks and noted that a few individuals did well on the production tasks and poorly on the perception tasks and vice versa. As scores were aggregated across all tasks, it is unknown if these patterns stem from both the melodic and rhythmic tasks or discrepancies in just one of the task categories.

We selected the BAT as our test of beat perception in part because of its use of real, readily accessible music, which is more likely to represent participants’ actual music listening experiences. The BAT successfully maintained most participants’ attention and engagement, but for some participants, the rich auditory information, with multiple instrumental timbres, may have interfered with their ability to perceive and consciously report on the beat. In comparison, the clapping tasks used in previous studies (Levitin and Bellugi, 1998; Martens et al., 2010) are presented in only one timbre. Thus, the sound complexity of the BAT stimuli may have exerted a greater effect on WS versus TD participants, especially given their poor auditory filtering and increased sensitivities to sounds, including to musical timbres (Levitin et al., 2005; John and Mervis, 2010; Lense et al., 2012). Additionally, though the BAT stimuli (especially with the exception of the Superman theme item) generally have low beat variability, they are not strictly isochronous as are stimuli in previous studies.

Furthermore, as correct/incorrect responses in production studies involving clapping were based on examiners’ judgments (Levitin and Bellugi, 1998; Martens et al., 2010), it is possible that more fine-grained analyses using acoustic measurements could have revealed group differences in either temporal precision or consistency. For example, raters did not perceive differences in the rhythmic accuracy of the singing of “Happy Birthday” by adults with WS versus chronological age-matched TD adults, but acoustic measurements demonstrated that the rhythmic patterns were less precise in the WS group (Martinez-Castilla and Sotillo, 2008). Additionally, Martens et al. (2010) noted that both WS and TD participants averaged above 90% on the clapping to the beat of the music task. Thus, a ceiling effect may have precluded finding group differences.

Beyond group differences, we found considerable individual variability in beat and meter perception skills in both the WS and TD groups. Similarly, previous studies with TD individuals have documented vast individual differences in beat perception (Iversen and Patel, 2008; Grahn and Schuit, 2012). Thus, it is not surprising to also find such broad individual differences in WS. Indeed, many participants with WS performed comparably to the TD group, including individuals scoring perfectly on the beat and/or meter measure, while others were at chance levels. IQ predicted beat and meter perception skills in the WS participants but not in the TD participants in our sample. This may be because of the wide variability in IQ in the WS participants while participants in the TD group all had IQ in the average range. Previous work in WS has indicated relationships between rhythm abilities and developmental level (e.g., Don et al., 1999). Synchronization of tapping behaviors to rhythmic stimuli also increases across development in TD children (e.g., Drake et al., 2000a).

In both WS and TD, beat and meter perception were associated with musical training. Our findings of the relationship between musical training and beat and meter skills in TD are consistent with previous studies (e.g., Drake et al., 2000a,b; Grahn and Schuit, 2012). Previous studies on musical timing perception (including rhythm, beat, and meter) in WS have not examined the role of musical training. However, studies of specific pitch perception abilities, singing skills, and musical instrument learning have all indicated a role of musical training in WS (Martinez-Castilla and Sotillo, 2008; Lense and Dykens, 2012; Lense et al., 2013). Therefore, the role of musical training in beat perception skills in WS appears to generally be consistent with findings in the TD population.

Even when controlling for IQ and musical training, sound processing style significantly predicted beat and meter performance in WS, and in the case of the BAT, was an even greater predictor than IQ. Previous studies in TD samples have not examined the role of sound processing style in beat perception, and we unfortunately were unable to collect this measure in our TD participants. However, greater use of a fundamental processing style in TD has been linked to preferences for playing percussion instruments (Schneider et al., 2005). Moreover, use of a fundamental processing style has been associated with a preference for hard rock music, which has a salient and consistent beat (while preference for jazz music, in contrast, is associated with greater use of spectral processing style; Schneider and Wengenroth, 2009). Individuals with WS exhibit a stronger fundamental processing style compared to TD individuals (Wengenroth et al., 2010). Additionally, individuals with WS primarily play percussion instruments (Lense et al., 2013) and listen to and prefer music with a strong and consistent beat such as hard rock or country rock/pop (Lense and Dykens, 2015).

The preferences for rock music in WS may therefore relate to sound processing style and a corresponding preference for music with a salient and consistent beat. Indeed, an item analysis on the BAT revealed that individuals with WS (but not TD individuals) had significant difficulty with one particular item, which was characterized by substantially greater beat variability than the other test items. It may seem surprising that beat variability did not predict accuracy in the TD group (or in WS after the exclusion of the outlier item) as temporal fluctuations influence beat perception and synchronization in TD individuals (Drake et al., 2000b; Large et al., 2002). However, the beat variability was generally quite low on the BAT items, particularly when excluding the Superman theme item. TD individuals are generally still able to track and synchronize to the beat in music despite temporal fluctuations (Large and Jones, 1999; Drake et al., 2000b). Thus, individuals with WS may have more difficulty finding and tracking the beat at lower thresholds of beat variability than TD individuals.

Once the Superman theme item was excluded, the range of beat variability in the stimuli was greatly reduced and beat variability no longer predicted item accuracy. However, controlling for tempo and beat variability, WS participants tended to do worse on orchestral pops than rock music excerpts, which may simply be a reflection of less exposure to orchestral music. In contrast, TD participants were more affected by tempo (performed better with faster items). This may be because the aligned/misaligned beeps always started 5 s into the musical track. Therefore, during faster tempo songs, participants would have heard more musical beats and thus may have better entrained to the beat of the songs to then determine the alignment of the superimposed beeps. It is not clear why the TD participants tended to do worse on the jazz items when controlling for other factors. It is possible that this genre of music was less familiar for the TD participants in our sample. While the BAT is a widely used measure of beat perception in TD, to our knowledge, thorough item level analyses have not been conducted. Future studies may want to carefully consider characteristics of the BAT stimuli when examining individual differences on this test or if choosing only a subset of items to administer.

Beyond factors that are typically thought of as being related to musical perception abilities (e.g., cognition, auditory processing, and musical training), Study 2 revealed that beat perception was significantly associated with adaptive Communication and Socialization skills, while meter perception was related to adaptive Socialization skills. These relationships, reflecting a medium effect size (Cohen, 1988), were maintained even when controlling for IQ. In contrast, there was no relationship with Daily Living Skills suggesting that beat and meter perception are specifically related to social communication abilities.^1^

While many studies have examined relationships between beat perception and specific linguistic and social tasks, to our knowledge, this is the first study to find relationships between beat perception and more global measures of social communication skills that reflect the performance of skills or behaviors in every day activities. Many of the skills that are aggregated in the Vineland-II have, however, been associated with beat perception in prior studies. For example, the Communication domain assesses Receptive (e.g., following directions), Expressive (e.g., conversations; grammatical skills), and Written (e.g., reading at a certain grade level) language abilities. Prior research has found that individual differences in beat perception relate to performance on standardized tasks of receptive and expressive language (e.g., Cumming et al., 2015) and word reading and reading comprehension (Corriveau and Goswami, 2009; Tierney and Kraus, 2013). Additionally, the presence of a beat-based or metric framework enhances speech comprehension (e.g., Rothermich and Kotz, 2013; Magne et al., 2016). Similarly, the Socialization domain assesses Interpersonal Relationships (e.g., understanding indirect cues, cooperating with others), Play and Leisure Time (e.g., following rules in games, going places with friends), and Coping (e.g., managing emotions, avoiding unsafe relationships) skills. Prior studies have indicated that musical beat-based activities promote cooperation (Anshel and Kippler, 1988; Wiltermuth and Heath, 2009; Kirschner and Tomasello, 2010; Cirelli et al., 2014) and feelings of connection (Demos et al., 2012).

Further support for the relationship between beat perception and social communication skills comes from neuroimaging studies. A network of auditory and motor processing areas contributes to beat perception involving the auditory cortex, supplementary motor area, premotor cortex, and basal ganglia (e.g., Grahn and McAuley, 2009; Grahn and Rowe, 2009). In particular, different components of the basal ganglia have been associated with beat prediction (Grahn and Rowe, 2009) and sensory timing (Schwartze et al., 2015), consistent with the involvement of the basal ganglia in prediction and prediction error more broadly. People with WS show reduced volume of the basal ganglia (Faria et al., 2012), and reduced volume or dysfunction of the basal ganglia has been associated with social difficulties in WS (Campbell et al., 2009) and autism (Qiu et al., 2010). Thus, general impairments in predictive timing may contribute to both musical beat perception and social communication difficulties, as successful prediction in a dynamic world is key to successful social engagement (e.g., Sinha et al., 2014). As a metric and beat-based stimulus, music may in part be appealing to individuals with WS because it provides a structured rhythmic framework that guides attention and increases predictability.

There are several limitations to the current study that should be noted. First, future studies should examine a variety of rhythm perception and production skills in the same group of individuals with WS to elucidate relationships among different aspects of temporal skills, including directly exploring relationships between perception and production and the role of beat perception in scaffolding rhythm perception in WS. We used real music that was not strictly isochronous but that generally had low levels of beat variability. Future studies could examine beat perception abilities along a wider spectrum of beat variability (including isochrony) to determine the range of variability to which individuals with WS versus TD are able to perceive and synchronize to a beat.

As well, we did not directly assess the role of movement in supporting beat perception. Consistent with task instructions, we asked participants to stay still during the BAT but encouraged them to move during the MBEA-m task. Informally, we observed that individuals who struggled the most on the MBEA-m task had no to minimal musical training and were least engaged in movement during these tasks. Additional work is needed on those individuals with WS who are unable to find the beat in music and move to it.

Finally, it will be important to address whether the relationship between social and communications skills and beat perception differ in WS compared with TD populations as studies in other domains (e.g., emotion) have found greater links between musical and social processing in WS than in TD (e.g., Lense et al., 2013; Ng et al., 2013). Relatedly, although we used a standardized measure of global communication and social skills (Vineland-II), future research could examine the role of beat perception in specific social communication tasks in WS and TD. TD individuals would be expected to score in the average range on a global measure such as the Vineland-II, which incorporates a variety of social communication skills. However, examination of specific social communication tasks with beat perception skills in WS and TD might reveal similarities and differences in specific, nuanced skills that contribute to successful social communication abilities. For example, TD individuals may be better able to compensate for a specific social communication weakness (e.g., by knowledge of how to act in a social situation) while this same vulnerability in WS might contribute to, or reflect, a broader constellation of social communication impairments. Furthermore, given the unique WS social profile of social communication deficits yet heightened social interest and motivation, it will be important to identify differences in beat perception in social versus non-social contexts in both WS and TD to examine how social context may differentially scaffold beat perception abilities in these populations.

Our finding that musical beat perception is related to social communication suggests that musical engagement and music therapy may be effective for social goals in WS. A growing number of studies report that group musical activities and/or music therapy promote social and emotional development in infants (Gerry et al., 2012), typically developing children with lower levels of prosocial behaviors (Schellenberg et al., 2015), and children with autism (Geretsegger et al., 2014). Increased musical interest in WS begins early in life (Levitin et al., 2004) and while music therapy is often appealing for WS given their musical interests, it is important to examine the mechanisms by which different musical experiences contribute to development in order to refine and optimize such interventions. For example, Martens et al. (2011) found that children with WS with versus without musical training did better on a verbal memory task involving novel words when it was administered via singing (to the tune of *Twinkle Little Star*) rather than speaking. It is likely that temporal structure differed across the singing versus speech conditions. In particular, the novel words in the singing condition all occurred on the musical beat. Children with musical training may have been more aware of the beat and metric structure of the song and thus may have been more attentive to the timing of the salient words. Of course, additional studies are needed that control for different aspects of the stimuli in order to test this hypothesis.

In summary, our study highlights the broad variability in musical beat and meter perception in WS and finds that these abilities are related to cognitive skills, sound processing style, and musical training. Moreover, we find relationships between beat and meter perception and social communication skills. To our knowledge, this is the first study to examine these relationships in a population with a unique social profile including social communication difficulties despite increased social motivation. Future studies are needed to determine if these relationships are also seen in other populations, or if this reflects closer links between predictive timing in musical and social contexts in WS.

## Author Contributions

ML and ED conceived and designed the experiments, ML conducted the experiments together with members of the lab, ML analyzed the data, ML and ED wrote the manuscript.

## Conflict of Interest Statement

The authors declare that the research was conducted in the absence of any commercial or financial relationships that could be construed as a potential conflict of interest.

## Footnotes

1 It is plausible that individuals with greater communication and social skills were simply better able to participate in the one-on-one testing session, leading them to perform better on the beat/meter perception tests. However, this seems unlikely for several reasons. First, all examiners were highly trained in working with individuals with WS and managing social difficulties. Additionally, a subset of 32 individuals also completed a pitch perception task during the same testing session with the same examiner. There was no relationship between pitch perception and any of the Vineland-II domains (ρ’s = -0.156 to -0.003, *p*’s > 0.3). Thus, adaptive social skills appear to be specifically related to beat/meter skills and not pitch skills.

## References

[B1] Anshel A. Kippler D. (1988). The influence of group singing on trust and cooperation. *J. Music Ther.* 25 145–155. 10.1093/jmt/25.3.145

[B2] Ayotte J. Peretz I. Hyde K. (2002). Congenital amusia: a group study of adults afflicted with a music-specific disorder. *Brain A J. Neurol.* 125(Pt 2), 238–251. 10.1093/brain/awf028 11844725

[B3] Campbell L. E. Daly E. Toal F. Stevens A. Azuma R. Karmiloff-Smith A. (2009). Brain structural differences associated with the behavioral phenotype in children with Williams syndrome. *Brain Res.* 1258 96–107. 10.1016/j.brainres.2008.11.101 19118537

[B4] Cirelli L. K. Einarson K. M. Trainor L. J. (2014). Interpersonal synchrony increases prosocial behavior in infants. *Dev. Sci.* 17 1003–1011. 10.1111/desc.12193 25513669

[B5] Cohen J. (1988). *Statistical Power Analysis for the Behavioral Sciences*, 2nd Edn Hillsdale, NJ: Lawrence Earlbaum Associates.

[B6] Corriveau K. H. Goswami U. (2009). Rhythmic motor entrainment in children with speech and language impairments: tapping to the beat. *Cortex* 45 119–130. 10.1016/j.cortex.2007.09.008 19046744

[B7] Cross I. (2003). Music and evolution: consequences and causes. *Contemp. Music Rev.* 22 79–89. 10.1080/0749446032000150906

[B8] Cumming R. Wilson A. Leong V. Colling L. J. Goswami U. (2015). Awareness of rhythmic patterns in speech and music in children with specific language impairments. *Front. Hum. Neurosci.* 9:672 10.3389/fnhum.2015.00672 PMC4686839 26733848

[B9] Demos A. P. Chaffin R. Begosh K. T. Daniels J. R. Marsh K. L. (2012). Rocking to the beat: effects of music and partner’s movements on spontaneous interpersonal coordination. *J. Exp. Psychol. Gen.* 141 49–53. 10.1037/a0023843 21668129

[B10] Dissanayake E. (2008). If music is the food of love, what about survival and reproductive success? *Musicae Sci. Special Issue* 12 169–195. 10.1177/1029864908012001081

[B11] Don A. J. Schellenberg E. G. Rourke B. P. (1999). Music and language skills of children with Williams syndrome. *Child Neuropsychol.* 5 154–170. 10.1076/chin.5.3.154.7337

[B12] Drake C. Jones M. R. Baruch C. (2000a). The development of rhythmic attending in auditory sequences: attunement, referent period, focal attending. *Cognition* 77 251–288. 10.1016/S0010-0277(00)00106-2 11018511

[B13] Drake C. Penel A. Bigand E. (2000b). Tapping in time with mechanically and expressively performed music. *Music Percept.* 18 1–23. 10.2307/40285899

[B14] Essens P. J. (1986). Hierarchical organization of temporal patterns. *Percept. Psychophys.* 40 69–73. 10.3758/BF03208185 3763362

[B15] Ewart A. K. Morris C. A. Atkinson D. Jin W. Sternes K. Spallone P. (1993). Hemozygosity at the elastin locus in a developmental disorder, Williams syndrome. *Nat. Genet.* 5 11–16. 10.1038/ng0993-11 7693128

[B16] Faria A. V. Landau B. O’Hearn K. M. Li X. Jiang H. Oishi K. (2012). Quantitative analysis of gray and white matter in Williams syndrome. *Neuroreport* 23 283–289. 10.1097/WNR.0b013e3283505b62 22410548 PMC3305911

[B17] Fleiss J. L. (1986). *The Design and Analysis of Clinical Experiments.* New York, NY: John Wiley and Sons.

[B18] Fujioka T. Trainor L. J. Large E. W. Ross B. (2012). Internalized timing of isochronous sounds in represented in neuromagnetic beta oscillations. *J. Neurosci.* 32 1791–1802. 10.1523/JNEUROSCI.4107-11.2012 22302818 PMC6703342

[B19] Geretsegger M. Elefant C. Mossler K. A. Gold C. (2014). Music therapy for people with autism spectrum disorder. *Cochrane Database. Syst. Rev.* 6:CD004381 10.1002/14651858.CD004381.pub3 PMC6956617 24936966

[B20] Gerry D. Unrau A. Trainor L. J. (2012). Active music classes in infancy enhance musical, communicative and social development. *Dev. Sci.* 15 398–407. 10.1111/j.1467-7687.2012.01142.x 22490179

[B21] Gordon E. E. (1986). *Primary Measures of Music Audiation.* Chicago: G.I.A. Publications.

[B22] Grahn J. A. Brett M. (2007). Rhythm and beat perception in motor areas of the brain. *J. Cogn. Neurosci.* 19 893–906. 10.1162/jocn.2007.19.5.893 17488212

[B23] Grahn J. A. Brett M. (2009). Impairment of beat-based rhythms discrimination in Parkinson’s disease. *Cortex* 45 54–61. 10.1016/j.cortex.2008.01.005 19027895

[B24] Grahn J. A. McAuley J. D. (2009). Neural bases of individual differences in beat perception. *Neuroimage* 47 1894–1903. 10.1016/j.neuroimage.2009.04.039 19376241

[B25] Grahn J. A. Rowe J. B. (2009). Feeling the beat: premotor and striatal interactions in musicians and nonmusicians during beat perception. *J. Neurosci.* 29 7540–7548. 10.1523/JNEUROSCI.2018-08.2009 19515922 PMC2702750

[B26] Grahn J. A. Schuit D. (2012). Individual differences in rhythmic ability: behavioral and neuroimaging investigations. *Psychomusicology* 22 105–121. 10.1037/a0031188

[B27] Green D. M. Swets J. A. (1966). *Signal Detection Theory and Psychophysics.* New York, NY: Wiley.

[B28] Grube M. Griffiths T. D. (2009). Metricality-enhanced temporal encoding and the subjective perception of rhythmic sequences. *Cortex* 45 72–79. 10.1016/j.cortex.2008.01.006 19058797

[B29] Honing H. (2013). “The structure and interpretation of rhythm in music,” in *Psychology of Music*, 3rd Edn, ed. Deutsch D. (London: Academic Press), 369–404. 10.1016/B978-0-12-381460-9.00009-2

[B30] Hopyan T. Dennis M. Weksberg R. Cytrynbaum C. (2001). Music skills and the expressive interpretation of music in children with Williams-Beuren syndrome: pitch, rhythm, melodic imagery, phrasing, and musical affect. *Child Neuropsychol. J. Normal Abnormal Dev. Childhood Adolesc.* 7 42–53. 10.1076/chin.7.1.42.3147 11815880

[B31] Iversen J. R. Patel A. D. (2008). “the beat alignment test (BAT): surveying beat processing abilities in the general population,” in *Proceedings of the 10th International Conference on Music Perception & Cognition (ICMPC10), August 2008*, Sapporo.

[B32] Jarvinen A. M. Bellugi U. (2013). What does Williams syndrome reveal about the determinants of social behavior? *Front. Hum. Neurosci.* 7:321 10.3389/fnhum.2013.00321 PMC3695384 23825455

[B33] John A. E. Mervis C. B. (2010). Sensory modulation impairments in children with Williams syndrome. *Am. J. Med. Gen. Part C Semin. Med. Gen.* 154C, 266–276. 10.1002/ajmg.c.30260 PMC2997471 20425786

[B34] Kaufman A. Kaufman N. (2004). *Kaufman Brief Intelligence Test*, 2nd Edn Circle Pines, MN: American Guidance Service.

[B35] Kirschner S. Tomasello M. (2010). Joint music making promotes prosocial behavior in 4-year-old children. *Evol. Hum. Behav.* 31 354–364. 10.1016/j.evolhumbehav.2010.04.004

[B36] Large E. Palmer C. (2002). Perceiving temporal regularity in music. *Cogn. Sci.* 26 1–37. 10.1207/s15516709cog2601_1

[B37] Large E. W. Fink P. Kelso J. A. (2002). Tracking simple and complex sequences. *Psychol. Res.* 66 3–17. 10.1007/s004260100069 11963276

[B38] Large E. W. Jones M. R. (1999). The dynamics of attending: hoe people track time-varying events. *Psychol. Rev.* 106 119–159. 10.1037/0033-295X.106.1.119

[B39] Laws G. Bishop D. (2004). Pragmatic language impair- ment and social deficits inWilliams syndrome: a comparison with Down’s syndrome and specific language impairment. *Int. J. Lang. Commun. Disord.* 39 45–64. 10.1080/13682820310001615797 14660186

[B40] Lense M. D. Dankner N. Pryweller J. R. Thornton-Wells T. A. Dykens E. M. (2014a). Neural correlates of amusia in Williams syndrome. *Brain Sci.* 4 594–612. 10.3390/brainsci4040594 25422929 PMC4279144

[B41] Lense M. D. Dykens E. M. (2012). Musical learning in children and adults with Williams syndrome. *J. Intell. Disab. Res.* 57 850–860. 10.1111/j.1365-2788.2012.01611.x 22974236

[B42] Lense M. D. Dykens E. M. (2015). “Beat perception, musical preferences, and sociability in Williams syndrome,” in *Poster session presented at the Society for Music Perception and Cognition (SMPC)*, Nashville, TN.

[B43] Lense M. D. Gordon R. L. Key A. P. Dykens E. M. (2012). “Neural correlates of musical timbre perception in Williams syndrome,” in *Proceedings of the 12th International Conference on Music Perception & Cognition (ICMPC12), July 2012*, Thessaloniki.

[B44] Lense M. D. Gordon R. L. Key A. P. Dykens E. M. (2014b). Neural correlates of affective priming with music in Williams syndrome. *Soc. Cogn. Affect. Neurosci.* 9 529–537. 10.1093/scan/nst017 23386738 PMC3989136

[B45] Lense M. D. Shivers C. M. Dykens E. M. (2013). (A)musicality in Williams syndrome: examining relationships among auditory perception, musical skill, and emotional responsiveness to music. *Front. Psychol.* 4:525 10.3389/fpsyg.2013.00525 PMC3744910 23966965

[B46] Levitin D. J. (2005). Musical behavior in a neurogenetic developmental disorder, Evidence from Williams syndrome. *Ann. N. Y. Acad. Sci.* 1060 1–10. 10.1196/annals.1360.027 16597782

[B47] Levitin D. J. Bellugi U. (1998). Musical Abilities in Individuals with Williams syndrome. *Music Percept.* 15 357–389. 10.2307/40300863

[B48] Levitin D. J. Cole K. Chiles M. Lai Z. Lincoln A. Bellugi U. (2004). Characterizing the musical phenotype in individuals with Williams Syndrome. *Child Neuropsychol.* 10 223–247. 10.1080/09297040490909288 15621847

[B49] Levitin D. J. Cole K. Lincoln A. Bellugi U. (2005). Aversion, awareness, and attraction: investigating claims of hyperacusis in the Williams syndrome phenotype. *J. Child Psychol. Psychiatry* 46 514–523. 10.1111/j.1469-7610.2004.00376.x 15845131

[B50] Loui P. Guenther F. H. Mathys C. Schlaug G. (2008). Action-perception mismatch in tone-deafness. *Curr. Biol.* 18 R331–R332. 10.1016/j.cub.2008.02.045 18430629 PMC2791531

[B51] Lovie P. (2005). “Coefficient of variation,” in *Encyclopedia of Statistics in Behavioral Science* Vol. 1 eds Everitt B. S. Howell D. C. (Chichester: John Wiley & Sons, Ltd.), 317–318.

[B52] Magne C. Jordan D. K. Gordon R. L. (2016). Speech rhythm sensitivity and musical aptitude: ERPs and individual differences. *Brain Lang.* 15 13–19. 10.1016/j.bandl.2016.01.001 26828758

[B53] Martens M. A. Jungers M. K. Steele A. L. (2011). Effect of musical experience on verbal memory in Williams syndrome: evidence from a novel word learning task. *Neuropsychologia* 49 3093–3102. 10.1016/j.neuropsychologia.2011.07.016 21807007

[B54] Martens M. A. Reutens D. C. Wilson S. J. (2010). Auditory cortical volumes and musical ability in Williams syndrome. *Neuropsychologia* 48 2602–2609. 10.1016/j.neuropsychologia.2010.05.007 20457168

[B55] Martens M. A. Wilson S. J. Reutens D. C. (2008). Research review: Williams syndrome: a critical review of the cognitive, behavioral, and neuroanatomical phenotype. *J. Child Psychol. Psychiatry* 49 576–608. 10.1111/j.1469-7610.2008.01887.x 18489677

[B56] Martinez-Castilla P. Sotillo M. (2008). Singing abilities in Williams syndrome. *Music Percept. Interdiscipl. J.* 25 449–469. 10.1525/mp.2008.25.5.449

[B57] Masataka N. (2001). Why early linguistic milestones are delayed in children with Williams syndrome: late onset of hand banging as a possible rate-limiting constraint on the emergence of canonical babbling. *Dev. Sci.* 4 158–164. 10.1111/1467-7687.00161

[B58] Mervis C. B. Becerra A. M. (2007). Language and communicative development in Williams syndrome. *Ment. Retard. Dev. Disabil. Res. Rev.* 13 3–15. 10.1002/mrdd.20140 17326109

[B59] Ng R. Lai P. Levitin D. J. Bellugi U. (2013). Musicality correlates with sociability and emotionality in Williams syndrome. *J. Ment. Health Res. Intellect. Disabil.* 6 268–279. 10.1080/19315864.2012.683932 24151530 PMC3799913

[B60] Patel A. D. (2006). Musical rhythm, linguistic rhythm, and human evolution. *Music Percept.* 24 99–104. 10.1037/a0032345

[B61] Patel A. D. (2008). *Music, Language, and the Brain.* New York, NY: Oxford University Press.

[B62] Patel A. D. Iversen J. R. Bregman M. R. Schulz I. (2009). Studying synchronization to a musical beat in nonhuman animals. *Ann. N. Y. Acad. Sci.* 1169 459–469. 10.1111/j.1749-6632.2009.04581.x 19673824

[B63] Patel A. D. Iversen J. R. Chen Y. Repp B. H. (2005). The influence of metricality and modality on synchronization with a beat. *Exp. Brain Res.* 163 226–238. 10.1007/s00221-004-2159-8 15654589

[B64] Peretz I. Champod A. S. Hyde K. (2003). Varieties of musical disorders. The Montreal Battery of Evaluation of Amusia. *Anna. N. Y. Acad. Sci.* 999 58–75. 10.1196/annals.1284.006 14681118

[B65] Plesa Skwerer D. Schofield C. Verbalis A. Faja S. Tager-Flusberg H. (2007). Receptive prosody in adolescents and adults with Williams syndrome. *Lang. Cogn. Process.* 22 247–271. 10.1080/01690960600632671

[B66] Pridmore M. Magne C. Gordon R. Dykens E. Lense M. Key A. (2014). “Affective priming effect of music on emotional prosody in Williams Syndrome,” in *Proceedings of the Fifth International Congress of Neurosciences, and Music*, Dijon.

[B67] Qiu A. Adler M. Crocetti D. Miller M. I. Mostofsky S. H. (2010). Basal ganglia shapes predict social, communication, and motor dysfunction in boys with autism spectrum disorder. *J. Am. Acad. Child Adolesc. Psychiatry* 49 539–551. 10.1016/j.jaac.2010.02.012 20494264

[B68] Rhodes S. M. Riby D. M. Fraser E. Campbell L. E. (2011). The extent of working memory deficits associated with Williams syndrome: exploration of verbal and spatial domains and executively controlled processes. *Brain Cogn.* 77 208–214. 10.1016/j.bandc.2011.08.009 21889249

[B69] Rothermich K. Kotz S. A. (2013). Prediction in speech comprehension: fMRI evidence on the meter-semantic interface. *Neuroimage* 70 89–100. 10.1016/j.neuroimage.2012.12.013 23291188

[B70] Schellenberg E. G. Corrigal K. A. Dys S. P. Malti T. (2015). Group music training and children’s prosocial skills. *PLoS ONE* 10:e0141449 10.1371/journal.pone.0141449 PMC4624672 26506414

[B71] Schneider P. Sluming V. Roberts N. Bleeck S. Rupp A. (2005). Structural, functional, and perceptual differences in Heschl’s gyrus and musical instrument preference. *Anna. N. Y. Acad. Sci.* 1060 387–394. 10.1196/annals.1360.033 16597790

[B72] Schneider P. Wengenroth M. (2009). The neural basis of individual holistic and spectral sound perception. *Contemp. Music Rev.* 28 315–328. 10.1080/07494460903404402

[B73] Schwartze M. Stockert A. Kotz S. A. (2015). Striatal contributions to sensory timing: voxel-based lesion mapping of electrophysiological markers. *Cortex* 71 332–340. 10.1016/j.cortex.2015.07.016 26298502

[B74] Sinha P. Kjelgaard M. M. Gandhi T. K. Tsourides K. Cardinaux A. L. Pantazis D. (2014). Autism as a disorder of prediction. *Proc. Natl. Acad. Sci. U.S.A.* 111 15220–15225. 10.1073/pnas.1416797111 25288765 PMC4210351

[B75] Sparrow S. S. Cicchetti D. V. Balla D. A. (2005). *Vineland Adaptive Behavior Scales*, 2 Edn Circle Pines, MN: American Guidance Service.

[B76] Stojanovik V. (2006). Social interaction deficits and conver- sational inadequacy in Williams syndrome. *J. Neurolinguist.* 19 157–173. 10.1016/j.jneuroling.2005.11.005

[B77] Thurman A. E. Fisher M. H. (2015). The Williams syndrome social phenotype: disentangling the contributions of social interest and social difficulties. *Int. Rev. Res. Dev. Disabil.* 49 191–227. 10.1016/bs.irrdd.2015.06.002

[B78] Tierney A. T. Kraus N. (2013). The ability to tap to a beat relates to cognitive, linguistic, and perceptual skills. *Brain Lang.* 124 225–231. 10.1016/j.bandl.2012.12.014 23400117 PMC3594434

[B79] Trevarthen C. (1999–2000). Musicality and the intrinsic motive pulse: evidence from human psychobiology and infant communication. *Musicae Sci. Special Issue* 3 155–215. 10.1177/10298649000030S109

[B80] Wengenroth M. Blatow M. Bendszus M. Schneider P. (2010). Leftward lateralization of auditory cortex underlies holistic sound perception in Williams Syndrome. *PLoS ONE* 5:e12326 10.1371/journal.pone.0012326 PMC2925895 20808792

[B81] Wiltermuth S. S. Heath C. (2009). Synchrony and cooperation. *Psychol. Sci.* 20 1–5. 19152536 10.1111/j.1467-9280.2008.02253.x

